# Normal Activation of Discoidin Domain Receptor 1 Mutants with Disulfide Cross-links, Insertions, or Deletions in the Extracellular Juxtamembrane Region: MECHANISTIC IMPLICATIONS*

**DOI:** 10.1074/jbc.M113.536144

**Published:** 2014-03-26

**Authors:** Huifang Xu, Takemoto Abe, Justin K. H. Liu, Irina Zalivina, Erhard Hohenester, Birgit Leitinger

**Affiliations:** From the ‡ National Heart and Lung Institute, Imperial College London, London SW7 2AZ, United Kingdom and; the § Department of Life Sciences, Imperial College London, London SW7 2AZ, United Kingdom

**Keywords:** Collagen, Mutagenesis Site-specific, Receptor Regulation, Receptor Structure-Function, Receptor Tyrosine Kinase, Signal Transduction, Activation Mechanism, Cysteine Scanning

## Abstract

The discoidin domain receptors, DDR1 and DDR2, are receptor tyrosine kinases that are activated by collagen. DDR activation does not appear to occur by the common mechanism of ligand-induced receptor dimerization: the DDRs form stable noncovalent dimers in the absence of ligand, and ligand-induced autophosphorylation of cytoplasmic tyrosines is unusually slow and sustained. Here we sought to identify functionally important dimer contacts within the extracellular region of DDR1 by using cysteine-scanning mutagenesis. Cysteine substitutions close to the transmembrane domain resulted in receptors that formed covalent dimers with high efficiency, both in the absence and presence of collagen. Enforced covalent dimerization did not result in constitutive activation and did not affect the ability of collagen to induce receptor autophosphorylation. Cysteines farther away from the transmembrane domain were also cross-linked with high efficiency, but some of these mutants could no longer be activated. Furthermore, the extracellular juxtamembrane region of DDR1 tolerated large deletions as well as insertions of flexible segments, with no adverse effect on activation. These findings indicate that the extracellular juxtamembrane region of DDR1 is exceptionally flexible and does not constrain the basal or ligand-activated state of the receptor. DDR1 transmembrane signaling thus appears to occur without conformational coupling through the juxtamembrane region, but requires specific receptor interactions farther away from the cell membrane. A plausible mechanism to explain these findings is signaling by DDR1 clusters.

## Teaser

**Background:** DDR1 is a constitutively dimeric receptor tyrosine kinase that is activated by collagen. The mechanism of transmembrane signaling is unknown.

**Results:** DDR1 activation is unaffected by disulfide cross-links, insertions, or deletions in the extracellular juxtamembrane region.

**Conclusion:** The extracellular juxtamembrane region of DDR1 does not transmit a conformational change across the cell membrane.

**Significance:** The activation mechanism of DDR1 appears to be unique among receptor tyrosine kinases.

## Introduction

Receptor tyrosine kinases (RTKs)^2^ are key regulators of fundamental cell functions such as proliferation and differentiation, cell survival, cell migration, and cell cycle control (1). These single-pass transmembrane (TM) proteins share a conserved cytosolic kinase domain but are structurally diverse in their extracellular ligand binding regions. The 58 human RTKs are divided into 20 subfamilies based on their extracellular domain architecture. RTK activity is tightly controlled in normal physiological conditions; dysregulation of RTK expression or function is associated with many pathologies, and drugs against unwanted RTK activation are in clinical use, in particular for cancer therapy (2). A paradigm for RTK activation emerged from experiments conducted in the 1980s: ligand binding causes receptor dimerization, which favors the autophosphorylation of key tyrosine residues in the activation loop of the catalytic kinase domains, resulting in kinase activation (3). The mechanisms of ligand-induced ectodomain dimerization differ between RTKs (1). Similarly, different strategies are used to keep the catalytic domains in an autoinhibited, inactive state in the absence of ligand (1). Typical RTKs (as exemplified by EGF, PDGF, or insulin receptor families) bind soluble growth factors and respond to ligand binding with autophosphorylation within seconds to minutes.

The discoidin domain receptors, DDR1 and DDR2, are a unique RTK family in that their ligands are collagens, which are key structural proteins in all types of extracellular matrix. Both DDRs are activated by a number of collagens (4–6) and play important roles in embryo development, with DDR1 being essential for mammary gland development (7) and DDR2 for the growth of long bones (8–10). Whereas the DDRs functionally overlap with other RTKs in controlling cellular processes such as cell migration, cell survival, differentiation, and proliferation, they additionally contribute to tissue homeostasis by regulating extracellular matrix remodeling (11–13). Dysregulation of DDR expression and/or function is linked to disease progression of a number of human diseases, including atherosclerosis, arthritis, organ fibrosis, and many types of cancer (11, 13, 14). Because the DDRs are deemed to play positive roles in pathologies, the use of DDR inhibitors is an attractive therapeutic approach.

The DDRs are distinguished from typical RTKs by several unusual, and poorly understood, characteristics. One of the unresolved puzzles is the slow activation kinetics of DDRs: maximal receptor activation (phosphorylation) is often achieved only hours after collagen stimulation and can remain detectable for more than a day (5, 6). Another feature that distinguishes the DDRs from most RTKs is that they form stable noncovalent dimers in the absence of ligand (15, 16). Therefore, it is difficult to envisage how the paradigm of ligand-induced receptor dimerization could apply to the DDRs. DDR dimerization seems to involve multiple contacts in the extracellular, TM, and cytosolic regions, with the TM region providing a key dimer interface (15). Using a bacterial TOXCAT reporter assay (17), we showed that the isolated DDR1 TM helices associate very strongly via a leucine-based sequence motif (15). A later systematic study confirmed the high self-association potential for the DDR1 and DDR2 TM domains, which gave the strongest signals of all RTKs in the TOXCAT assay (18).

DDR1 and DDR2 have the same domain organization. Their extracellular regions are composed of two globular domains, the N-terminal discoidin (DS) domain and the DS-like domain, followed by a large juxtamembrane (JM) region (19). In DDR1, this JM region consists of 50 amino acid residues and is rich in glycine and proline residues, suggesting that it may be unstructured (20, 21). The cytosolic regions also contain a large JM region (up to 171 amino acid residues in DDR1, depending on the isoform) before the C-terminal catalytic kinase domain. The collagen binding site is entirely contained in the DS domain (4, 22), which adopts an 8-stranded β-barrel structure (19, 22, 23). The DDRs recognize specific amino acid motifs in fibrillar collagens (24, 25), which bind to a conserved trench at the top of the DS domain (19, 22, 23). The structures of the unliganded DDR1 DS domain (19) and the collagen-bound DDR2 DS domain (23) are very similar and offer no clues as to how ligand binding to the DS domain leads to activation of the intracellular kinase domain. This contrasts sharply with the situation of collagen-binding integrins, where large scale changes in the collagen binding domain are linked to conformational changes in the rest of the receptor that drive transmembrane signaling (26).

We previously hypothesized that conformational changes within a DDR dimer may cause cytosolic kinase activation (15). Following usage in the EGF receptor field, we use the term “conformational coupling” to describe the series of defined structural changes that link ligand binding to kinase activation within a receptor dimer (27, 28). In an effort to define functionally important dimer contacts in DDR1, we used cysteine-scanning mutagenesis, a powerful technique that has been used to define the active conformations of other cell surface receptors (*e.g.* Refs. 29–32). Using this method, covalent dimerization by disulfide bridges is enforced at various positions, which allows conclusions to be drawn about the relative geometry of receptor protomers within signaling dimers. For some receptors, cysteine substitution mutagenesis leads to constitutively active mutants (29, 30, 33, 34), whereas for other receptors regions that are involved in conformational changes can be identified (32). We found that the 10 extracellular JM residues of DDR1 closest to the TM domain could be cross-linked with very high efficiency. Remarkably, collagen stimulation did not affect cross-linking efficiency, and the covalent cross-links did not affect collagen-induced receptor activation. Insertions of flexible segments into the JM region or deletions within the JM region were also tolerated without affecting DDR1 activation. Thus, transmembrane signaling of DDRs does not appear to involve conformational coupling through the JM region. In contrast, cross-links farther away from the TM domain markedly reduced collagen-induced receptor phosphorylation, suggesting that specific contacts between the extracellular regions are required for DDR1 activation, plausibly in the context of receptor clusters.

## EXPERIMENTAL PROCEDURES

### 

#### 

##### Cell Culture

Human embryonic kidney (HEK) 293 cells (ATCC, Manassas, VA) were grown in Dulbecco's modified Eagle's medium/F12 nutrient mixture (Invitrogen) supplemented with 2 mm l-glutamine and 10% fetal bovine serum at 37 °C, 5% CO_2_.

##### Chemicals and Reagents

Collagen I (acid-soluble from rat tail; C-7661) was from Sigma. *N*-Ethylmaleimide (NEM) was purchased from Sigma (E-1271). The antibodies (Abs) and their sources were as follows: rabbit-anti-DDR1 (SC-532) from Santa Cruz Biotechnology; mouse anti-phosphotyrosine, clone 4G10, from Upstate Biotechnology; rabbit anti-DDR1 (phosphotyrosine 513) from Abbexa Ltd. (Cambridge, UK); goat anti-rabbit Ig horseradish peroxidase-conjugated (P0448, DAKO A/S, Denmark); sheep anti-mouse Ig-horseradish peroxidase (Amersham Biosciences); and goat anti-mouse IgG FITC-conjugated (F-9006, Sigma). Mouse anti-DDR1 monoclonal Abs were generated in our laboratory (19). Anti-DDR1 (phosphotyrosine 513), here referred to as anti-pY513, is a phospho-specific Ab that recognizes phosphorylated Tyr-513 in DDR1b. Specificity for phosphorylated DDR1b, and not DDR1a, was confirmed (data not shown). Rabbit anti-pY-DDR1 (pY792/796/797), a custom-made Ab against the triple-phosphorylated DDR1 activation loop, was made by Abnova Taiwan Corporation, using as immunogen a peptide encompassing amino acids 780–799 of DDR1 (DDR1b nomenclature). The antigen was KLH-CIKIADFGMSRNL(pY)AGD(pY)(pY)RV.

##### DNA Constructs and Site-directed Mutagenesis

All cysteine substitution mutants and the GS1 and GS2 constructs were generated by strand overlap extension PCR (4) using a cDNA encoding DDR1 C287S as a template. DDR1 C287S and the JM deletion mutants were created by the same method, using a cDNA encoding wild-type human DDR1b as template. The primer sequences used to generate the mutations are available on request. PCR products containing the relevant mutations were cut with SacI and XhoI and subcloned into a pGEM-3Z (Promega)-based vector encoding full-length DDR1b, after the corresponding wild-type sequence was removed. cDNAs encoding mutant DDR1b were cloned into the mammalian expression vector pRK5 (BD Biosciences). All PCR-derived DNA constructs were verified by DNA sequencing.

##### Transfection of HEK293 Cells

HEK293 cells were grown either in 24-well tissue culture plates (for cross-linking and autophosphorylation assays) or in 6-well plates (for flow cytometry). Cells were transfected with relevant DDR1 expression plasmids by calcium phosphate precipitation.

##### Cysteine Cross-linking Assay

24 h after transfection, cells were incubated with serum-free medium for 16 h. Cells were then stimulated with 10 μg/ml collagen I for 90 min at 37 °C and lysed in 1% Nonidet P-40, 150 mm NaCl, 50 mm Tris, pH 7.4, 1 mm EDTA, 1 mm phenylmethylsulfonyl fluoride, 50 μg/ml aprotinin, 1 mm sodium orthovanadate, 5 mm NaF, and 10 mm NEM. Aliquots of the lysates were analyzed by nonreducing SDS-PAGE on 5% polyacrylamide gels followed by blotting onto nitrocellulose membranes. The blots were probed with anti-DDR1 Abs followed by horseradish peroxidase-conjugated goat anti-rabbit secondary Abs. Signal detection was performed using Enhanced Chemiluminescence Plus reagent (Amersham Biosciences) on an Ettan DIGE Imager (GE Healthcare). Intensities of protein bands were quantitated using ImageQuant TL (GE Healthcare).

##### DDR1 Autophosphorylation Assay

The assay was performed as described before (4). Briefly, 24 h after transfection, the cells were incubated with serum-free medium for 16 h. Cells were then stimulated with 10 μg/ml collagen I for up to 90 min at 37 °C. Cells were lysed in 1% Nonidet P-40, 150 mm NaCl, 50 mm Tris, pH 7.4, 1 mm EDTA, 1 mm phenylmethylsulfonyl fluoride, 50 μg/ml aprotinin, 1 mm sodium orthovanadate, and 5 mm NaF. Aliquots of the lysates were analyzed by reducing SDS-PAGE on 7.5% polyacrylamide gels followed by blotting onto nitrocellulose membranes. The blots were probed first with anti-phosphotyrosine Abs followed by horseradish peroxidase-conjugated sheep anti-mouse or horseradish peroxidase-conjugated goat anti-rabbit secondary Abs, as required, then stripped in antibody stripping solution (Alpha Diagnostic International, San Antonio, TX) and reprobed with anti-DDR1 Abs followed by horseradish peroxidase-conjugated goat anti-rabbit secondary Abs.

##### Flow Cytometry

HEK293 cells in 6-well tissue culture plates were transfected with the relevant DDR1 expression plasmids and grown for 48 h before being dissociated with nonenzymatic cell dissociation solution (Sigma) and resuspended in PBS containing 1% BSA. The cells were incubated for 30 min on ice with mouse anti-DDR1 monoclonal Abs at 10 μg/ml in 100 μl of PBS/BSA. Cells were then washed three times with PBS/BSA and incubated with FITC-conjugated goat anti-mouse IgG for 30 min on ice. After three washes as above, the cells were resuspended in 2% formaldehyde in PBS. Data were collected on a BD LSRFortessa cell analyzer using BD FACSDiva software 6.0 (BD Biosciences) and further analyzed on FlowJo software 7.6.4 (Tree Star, Inc.).

## RESULTS

### 

#### 

##### Design of DDR1 Cysteine Mutations

**FIGURE 1.**
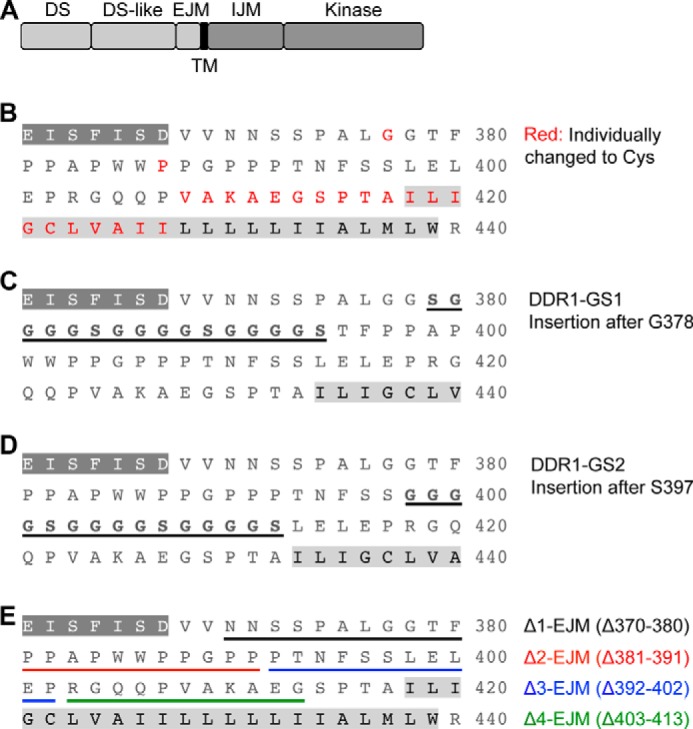
**DDR1 mutants used in this study.** *A*, DDR1 is composed of globular DS and DS-like domains followed by an extracellular juxtamembrane (*EJM*) region of 50 amino acids, a TM domain, an intracellular juxtamembrane (*IJM*) region, and a kinase domain. *B*, amino acid substitutions in the extracellular JM and TM regions. The C-terminal amino acids of the DS-like domain are shown as *white letters* on a *dark gray* background. The TM domain is highlighted in *light gray*. Amino acids that were substituted individually by cysteine are highlighted in *red. C*, insertion mutant DDR1-GS1. A 16-amino acid sequence was inserted after Gly-378. The inserted amino acids are in *bold* and *underlined. D*, insertion mutant DDR1-GS2. A 15-amino acid sequence was inserted after Ser-397. The inserted amino acids are in *bold* and *underlined. E*, deletion mutants. The deleted regions are highlighted in *different colors*, with the color scheme and amino acid deletions defined on the *right*.

In this study, we replaced individual amino acids of the extracellular JM region with cysteine (Fig. 1, *A* and *B*). The DDR1 extracellular region contains seven cysteines, six of which form disulfide bonds (19). The unpaired cysteine, Cys-287, is buried in the DS-like domain (19). To avoid potential cross-linking of the unpaired Cys-287, we performed all our cysteine mutagenesis in the C287S background. The C287S DDR1 construct was indistinguishable from wild-type DDR1 in terms of cell surface expression, dimerization, and activation by collagen (data not shown).

##### Cysteine Mutations in the JM Region Close to the Plasma Membrane

**FIGURE 2.**
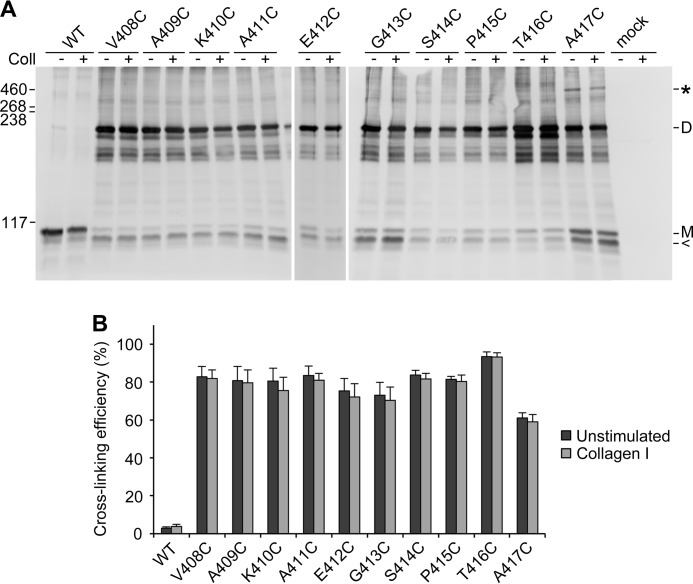
**Disulfide cross-linking of DDR1 JM cysteine mutants.** Transiently transfected HEK293 cells were stimulated with 10 μg/ml collagen I (*Coll*) for 90 min at 37 °C or left untreated. Cells were lysed in the presence of 30 mm NEM. Lysates were subjected to nonreducing SDS-PAGE on 5% polyacrylamide gels followed by Western blotting with anti-DDR1 antibodies. *A*, the positions of molecular mass markers (in kDa) are indicated on the *left. M* indicates mature monomeric DDR1, *D* indicates dimeric DDR1, < indicates intracellular DDR1. The *asterisk* indicates the position of higher order oligomers for the A417C mutant. *B*, quantitation of cross-linking efficiency, expressed as the percentage of dimeric DDR1 with respect to total DDR1. For A417C, cross-linking efficiency comprises both dimeric and higher oligomeric forms. The data are the means ± S.E. (*error bars*) of 3–6 independent experiments.

Because the DDR1 TM domain has a very high propensity for self-interactions (15, 18), we hypothesized that the JM region closest to the TM domain might also mediate dimer contacts. If this were the case, cysteine substitution of amino acids close to the TM domain should lead to covalent cross-linking by disulfide bonds, which can be detected by nonreducing SDS-PAGE. Cells were transfected with full-length DDR1 expression constructs and lysed in the presence of NEM to avoid potential nonnative disulfide formation during cell lysis. As expected, DDR1 C287S (referred to as wild-type from here on) did not show any covalent dimerization. In sharp contrast, we observed high levels of cross-linking for the 10 mutants in which amino acids close to the TM domain were individually replaced by cysteine (V408C to A417C) (Fig. 2). Mutants V408C to T416C showed dimerization efficiencies of >70%, with T416C being the most highly cross-linked mutant with >90% dimer. DDR1 A417C showed a somewhat lower dimerization efficiency, possibly because Ala-417 is on the border with, or already part of, the TM domain.

DDR1 migrates as two glycoforms on SDS-PAGE, with the upper molecular mass band corresponding to the mature, cell surface-expressed, receptor and the lower form to the biosynthetic precursor (15). As it is difficult to separately quantitate the upper and lower forms, we estimated the proportion of cross-linked dimers relative to total DDR1. Therefore, cross-linking of the mature form may be even more efficient than suggested by Fig. 2*B*. In fact, very little monomeric protein remained detectable for the mature forms of mutants V408C to T416C, indicating almost complete dimerization (Fig. 2*A*).

The ability to form a disulfide cross-link requires the residues to be closely apposed. Our results thus provide compelling evidence that DDR1 forms ligand-independent dimers (15). The high degree of dimerization of the cysteine mutants strongly suggests that DDR1 dimers are constitutive, rather than in a dynamic equilibrium with monomers. Apart from T416C showing a slightly higher cross-linking efficiency, there was no apparent pattern or periodicity in the cross-linking efficiencies of mutants V408C to T416C, indicating that the extracellular JM region of DDR1 does not adopt a defined secondary structure. The conformation of the JM region appears to be insensitive to ligand binding, given that treatment with collagen had no effect on the cross-linking efficiency (Fig. 2).

##### Cysteine Mutations in the TM Domain

**FIGURE 3.**
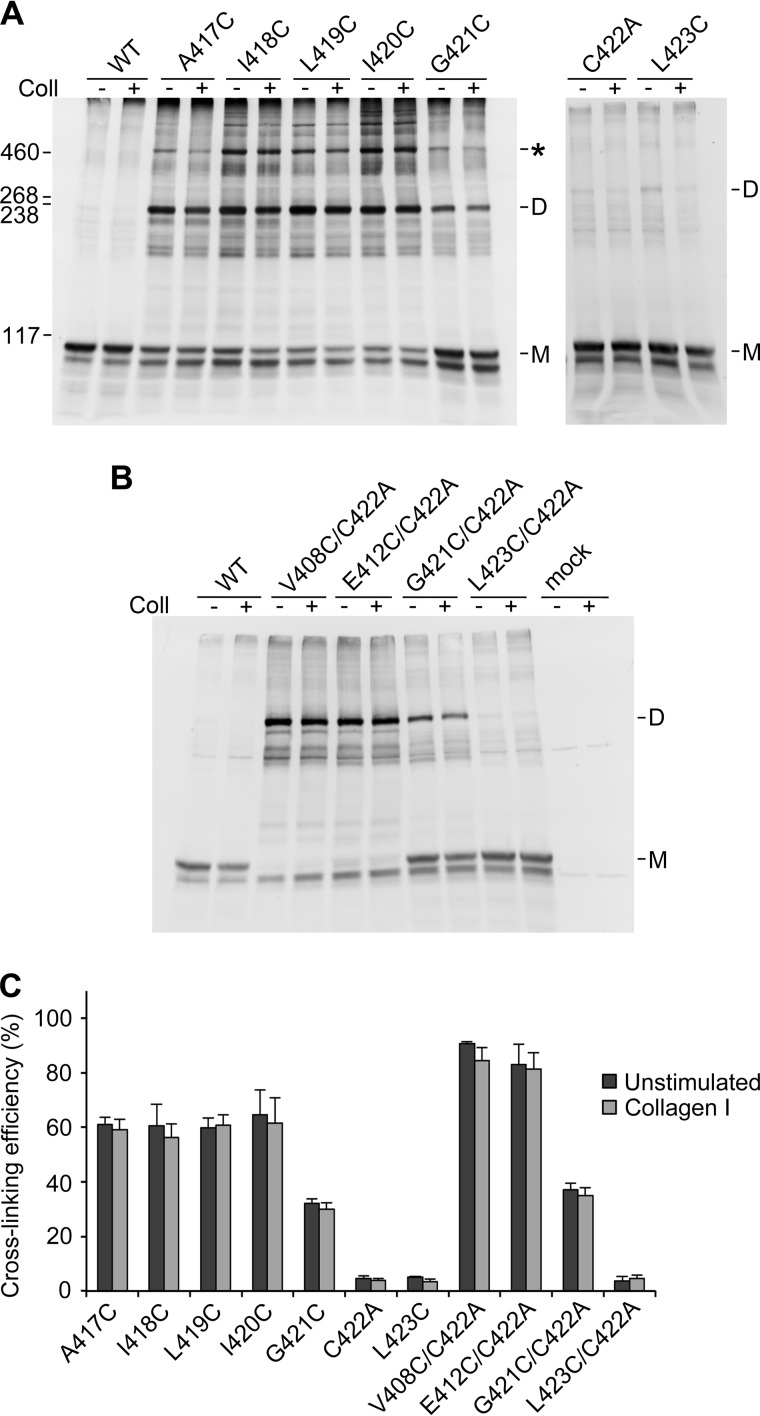
**Disulfide cross-linking of DDR1 TM cysteine mutants.** Transiently transfected HEK293 cells were stimulated with 10 μg/ml collagen I (*Coll*) for 90 min at 37 °C or left untreated. Cells were lysed in the presence of 30 mm NEM. Lysates were subjected to nonreducing SDS-PAGE on 5% polyacrylamide gels followed by Western blotting with anti-DDR1 antibodies. *A*, single mutants. The positions of molecular mass markers (in kDa) are indicated on the *left. M* indicates mature monomeric DDR1, *D* indicates dimeric DDR1. The *asterisk* indicates the position of higher order oligomers. *B*, double mutants. *C*, quantitation of cross-linking efficiency, expressed as the percentage of dimeric and higher oligomeric forms with respect to total DDR1. The data are the means ± S.E. (*error bars*) of 3–6 independent experiments.

We also replaced amino acids in the N-terminal part of the TM domain by cysteine and found that the most N-terminal residues (418–421) could be cross-linked (Fig. 3). Residue 422 of the DDR1 TM domain naturally is a cysteine, which does not form a disulfide bond (WT in Figs. 2*A* and 3*A*). Cysteine substitution at residue Leu-423 (Fig. 3) or the following residues (tested up to residue 427; data not shown) did not result in any cross-linking. This is most likely due to these cysteines being inaccessible to the enzymes in the endoplasmic reticulum required for disulfide bond formation (35).

To rule out that the unpaired Cys-422 in the TM domain is responsible for cross-linking of our mutants in the JM region, we made selected mutants in the C422A background (actually C287S/C422A). V408C/C422A and E412C/C422A showed slightly increased dimerization efficiencies compared with V408C and E412C, respectively, ruling out that Cys-422 is responsible for covalent cross-linking of these mutants. However, we noticed that TM mutants with cross-linking ability displayed an additional high molecular mass band, which migrated at a position consistent with tetramer formation and was not observed in any of the JM mutants (marked by *asterisks* in Figs. 2*A* and 3*A*). This band could conceivably arise from receptors that are cross-linked by Cys-422 in addition to the TM residue substituted by cysteine. This notion is supported by the observation that the G421C/C422A double mutant, unlike the G421C mutant, did not show the high molecular mass band (Fig. 3*B*). Thus, the exposure and/or reactivity of Cys-422 may be altered in the TM domain mutants.

##### Covalent Cross-linking in the JM or TM Regions Does Not Affect Receptor Activation

**FIGURE 4.**
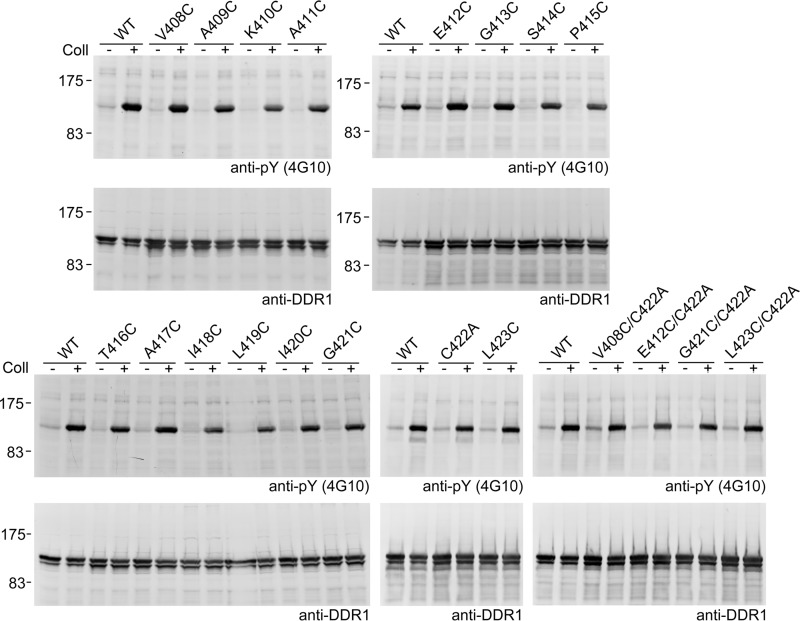
**Autophosphorylation of DDR1 JM and TM cysteine mutants.** Transiently transfected HEK293 cells were stimulated with 10 μg/ml collagen I (*Coll*) for 90 min at 37 °C or left untreated. Cells were lysed, and lysates were analyzed by reducing SDS-PAGE on 7.5% polyacrylamide gels and Western blotting. The blots were probed with anti-phosphotyrosine (*anti-pY*) monoclonal antibody 4G10 (*upper blots*), followed by stripping and reprobing with anti-DDR1 antibodies (*lower panels*). The positions of molecular mass markers are indicated (in kDa). The data are representative of ≥3 independent experiments.

We expected the enforced covalent dimerization by disulfide bridges to result in either constitutively active or inactive DDR1 mutants. To our surprise, none of the JM and TM cysteine substitution mutants was constitutively active, and all of them showed collagen-induced autophosphorylation at levels comparable with the levels exhibited by wild-type DDR1 (Fig. 4). Thus, covalently locking the DDR1 dimers at different positions close to or within the plasma membrane does not impair receptor activation.

##### Cysteine Mutations Farther Away from the Plasma Membrane: JM Region

**FIGURE 5.**
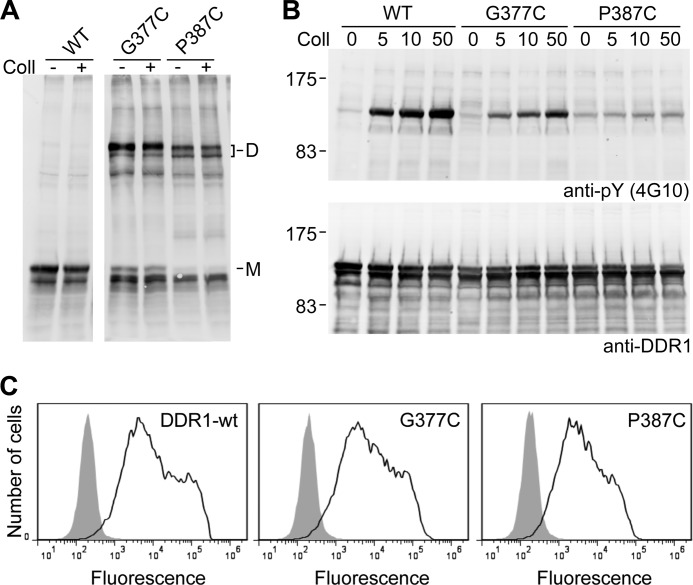
**Disulfide cross-linking and autophosphorylation of G377C and P387C mutants.** *A*, transiently transfected HEK293 cells were stimulated with 10 μg/ml collagen I (*Coll*) for 90 min at 37 °C or left untreated. Cells were lysed in the presence of 30 mm NEM. Lysates were subjected to nonreducing SDS-PAGE on 5% polyacrylamide gels followed by Western blotting with anti-DDR1 antibodies. *M* indicates mature monomeric DDR1, *D* indicates dimeric DDR1. *B*, autophosphorylation. Cells were stimulated with the indicated concentrations of collagen I (in μg/ml) for 90 min at 37 °C. Cell lysates were analyzed by reducing SDS-PAGE on 7.5% polyacrylamide gels and Western blotting. The blot was probed with anti-phosphotyrosine (*anti-pY*) monoclonal antibody 4G10 (*upper blot*), followed by stripping and reprobing with anti-DDR1 antibodies (*lower blot*). The positions of molecular mass markers are indicated (in kDa). *C*, cell surface expression. Transiently transfected HEK293 cells were stained on ice with mouse anti-DDR1 monoclonal Ab 7A9 followed by FITC-conjugated secondary Abs and flow cytometry. *Filled gray histograms*, secondary Abs only; *open histograms*, anti-DDR1. The data are representative of ≥3 independent experiments.

We also tested two residues farther away from the TM domain and replaced Gly-377 and Pro-387 with cysteines. These two mutants showed efficient ligand-independent dimerization (Fig. 5*A*). Compared with wild-type DDR1 and the other JM mutants, the G377C and P387C mutants had higher proportions of biosynthetic intermediates (Fig. 5*B*), but flow cytometry confirmed that the mutants were present at the cell surface at comparable levels with wild-type DDR1 (Fig. 5*C*). Collagen induced DDR1 G377C autophosphorylation at concentrations similar to those for wild-type DDR1, but the mutant showed somewhat reduced phosphorylation signals. In contrast, the P387C mutation showed no collagen-induced phosphorylation response and instead displayed a low level of constitutive phosphorylation (Fig. 5*B*).

##### Cysteine Mutations Farther Away from the Plasma Membrane: DS-like Domain

**FIGURE 6.**
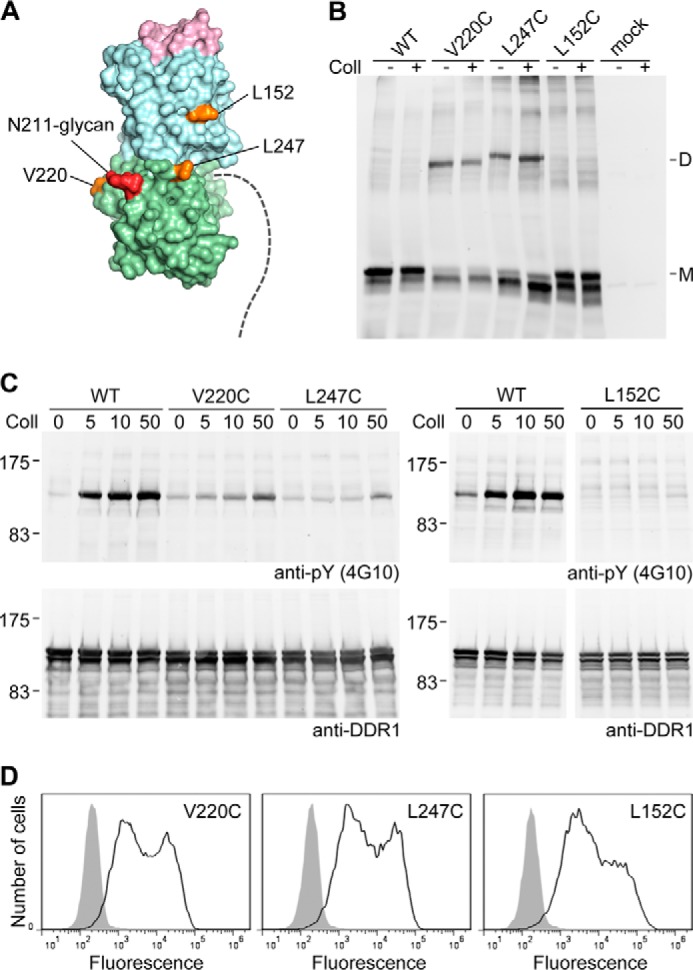
**Disulfide cross-linking and autophosphorylation of L152C, V220C, and L247C mutants.** *A*, location of mutated residues. Shown is a surface representation of DS (*light blue*) and DS-like (*light green*) domains of DDR1 (19). The collagen binding trench at the top of the DS domain is in *pink*. The three residues mutated to cysteine are in *orange* and labeled. Asn-211 and the innermost two sugar moieties that are visible in the x-ray structure are labeled in *red*. The last residue of the DS-like domain, Asp-367, is at the back in this view, and the start of the JM region is indicated by a *dashed line. B*, disulfide cross-linking. Transiently transfected HEK293 cells were stimulated with 10 μg/ml collagen I (*Coll*) for 90 min at 37 °C or left untreated. Cells were lysed in the presence of 30 mm NEM. Lysates were subjected to nonreducing SDS-PAGE on 5% polyacrylamide gels followed by Western blotting with anti-DDR1 antibodies. *M* indicates mature monomeric DDR1, *D* indicates dimeric DDR1. *C*, autophosphorylation. Cells were stimulated with the indicated concentrations of collagen I (in μg/ml) for 90 min at 37 °C. Cell lysates were analyzed by reducing SDS-PAGE on 7.5% polyacrylamide gels and Western blotting. The blot was probed with anti-phosphotyrosine (*anti-pY*) monoclonal antibody 4G10 (*upper blot*), followed by stripping and reprobing with anti-DDR1 antibodies (*lower blot*). The positions of molecular mass markers are indicated (in kDa). *D*, cell surface expression. Transiently transfected HEK293 cells were stained on ice with mouse anti-DDR1 monoclonal Ab 7A9 followed by FITC-conjugated secondary Abs and flow cytometry. *Filled gray histograms*, secondary Abs only; *open histograms*, anti-DDR1. The data are representative of ≥3 independent experiments.

Our results so far demonstrate that the DDR1 JM region, with the single exception of Pro-387, tolerates covalent cross-links without impact on receptor activation. We explored whether the flexibility of the JM region would allow residues of the DS-like domain to participate in covalent dimerization. Based on the crystal structure of the DDR1 DS and DS-like domains (19), we identified two residues on the protein surface that could conceivably cross-link when replaced with cysteines, Val-220 and Leu-247 (Fig. 6*A*). The V220C and L247C mutants formed disulfide-linked dimers (Fig. 6*B*) and were detectable at the cell surface (Fig. 6*D*). The electrophoretic mobility of cross-linked DDR1 showed some dependence on the distance of the disulfide bond from the end of the polypeptide chain, as noted for similar experiments on EGF receptor mutants (31). The DDR1 V220C and L247C mutants had strongly impaired collagen-induced autophosphorylation levels compared with wild-type DDR1 and displayed low levels of constitutive phosphorylation, like the P387C mutant (Fig. 6*C*). Thus, locking the DDR1 dimer at the level of the DS-like domain interferes with receptor activation.

##### Cysteine Mutation in the DS Domain

We previously identified a conserved patch on the surface of the DDR1 DS domain that is involved in TM signaling but does not participate in ligand binding (19). We replaced one of these residues, Leu-152, with cysteine. The L152C mutant was not able to form a disulfide-linked dimer (Fig. 6*B*), demonstrating the specificity of our cross-linking assay. Consistent with the established role of Leu-152 in TM signaling (19), the L152C mutant did not show collagen-induced receptor phosphorylation despite normal cell surface expression (Fig. 6, *C* and *D*). We previously showed that replacing Leu-152 with Glu does not affect collagen binding (19). It is therefore highly unlikely that the L152C mutant is inactive due to loss of collagen binding.

##### Insertions or Deletions in the JM Region

**FIGURE 7.**
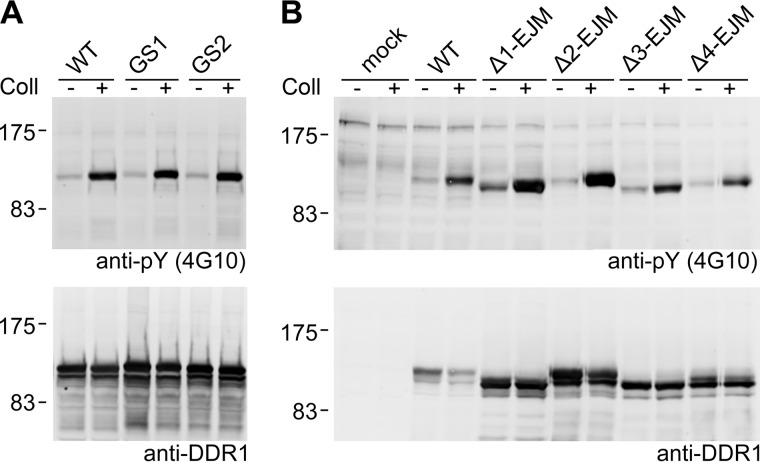
**Autophosphorylation of DDR1 mutants with insertions and deletions in the JM region.** *A*, insertion mutants. *B*, deletion mutants. Transiently transfected HEK293 cells were stimulated with 10 μg/ml collagen I (*Coll*) for 90 min at 37 °C or left untreated. Cells were lysed, and lysates were analyzed by reducing SDS-PAGE on 7.5% polyacrylamide gels and Western blotting. The blots were probed with anti-phosphotyrosine (*anti-pY*) monoclonal antibody 4G10 (*upper blots*), followed by stripping and re-probing with anti-DDR1 antibodies (*lower panels*). The positions of molecular mass markers are indicated (in kDa). The data are representative of ≥3 independent experiments.

To probe the role of the extracellular JM region in DDR1 activation using a different approach, we inserted flexible linkers of 15–16 amino acids into two separate locations and successively deleted four stretches of 11 amino acids (Fig. 1, *C–E*). All of these mutants responded to collagen stimulation with autophosphorylation similar to wild-type DDR1, further highlighting the extraordinary tolerance of the JM region to mutation (Fig. 7). In contrast to a previously generated JM deletion construct (15), the deletion mutants generated for the present study were all trafficked to the cell surface (data not shown).

##### Monitoring Specific Phosphorylation Sites

**FIGURE 8.**
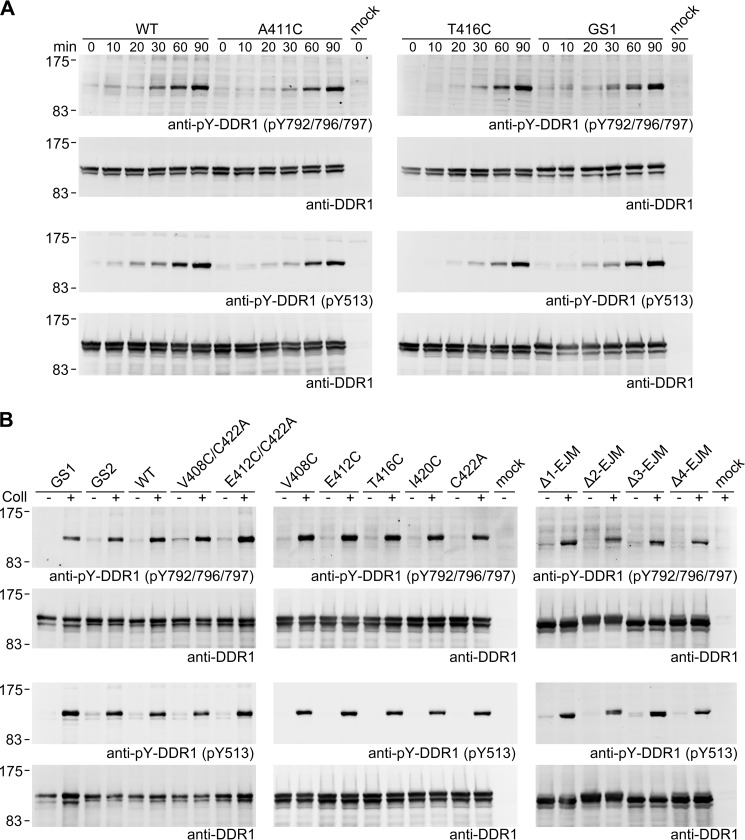
**Site-specific phosphorylation of selected DDR1 Cys mutants.** Transiently transfected HEK293 cells were stimulated at 37 °C with 10 μg/ml collagen I (*Coll*) for the indicated times (*A*) or for 90 min (*B*) or left untreated. Cells were lysed, and lysates were analyzed by reducing SDS-PAGE on 7.5% polyacrylamide gels and Western blotting. The blots were probed with phospho-specific Abs, either against the triple-phosphorylated DDR1 activation loop (*anti-pY-DDR1*, *pY792/796/797*) or against phosphotyrosine 513 in DDR1b (*anti-pY-DDR1*, *pY513*). The positions of molecular mass markers are indicated (in kDa). The data are representative of ≥2 independent experiments.

Our results show that covalent cross-linking of residues in the proximal JM or TM regions, as well as insertions or deletions within the JM region, do not affect DDR1 activation as monitored by total tyrosine phosphorylation (Figs. 4 and 7). These data, however, do not rule out differences in phosphorylation of specific cytoplasmic tyrosine residues. We therefore tested our DDR1 mutants for collagen-induced phosphorylation at specific sites. We monitored phosphorylation of the kinase activation loop (Tyr-792/796/797) and of Tyr-513, a residue present in the intracellular JM region of the DDR1b isoform, but not the DDR1a isoform. A time course of DDR1 phosphorylation detected activation loop and Tyr-513 phosphorylation with similar kinetics in wild-type DDR1 and selected mutants (Fig. 8*A*). In addition, all of the mutants underwent phosphorylation at both sites to similar extents as wild-type DDR1 when incubated with collagen I for 90 min (Fig. 8*B* and data not shown). Thus, neither enforced dimerization of the proximal JM region nor insertions or deletions within the JM region affect DDR1 activation as detected by our assays.

## DISCUSSION

Classical experiments with chimeric receptors suggested that many RTKs use a similar mechanism for signal transduction across the cell membrane. For example, a chimeric receptor containing the extracellular domain of insulin receptor and the TM and cytosolic domains of EGF receptor could be activated by insulin (36). Replacing the DDR1 ectodomain with that of the PDGF receptor did not result in a chimaera that could be activated by PDGF (37), giving the first indication that the mechanism of DDR activation might differ from that of prototypical RTKs. A unique mechanism is also suggested by the slow kinetics of DDR activation (5, 6).

In a previous study, we used chemical cross-linking and co-immunoprecipitation to show that the DDRs are dimers (or higher oligomers) in the absence of collagen (15). FRET data by others also indicated the presence of oligomeric DDR1 on the cell surface (16). In the present study, we found that cysteines introduced into the JM region of DDR1 formed disulfides with very high efficiency regardless of their position. Collagen did not change the level of dimerization observed, in agreement with our earlier study (15). The new findings provide compelling evidence that DDR1 indeed is a constitutive dimer. Because disulfide formation takes place in the endoplasmic reticulum, DDR1 dimerization must occur already during biosynthesis, a conclusion also drawn in our earlier study (15). In contrast to our results, cysteine mutagenesis of the EGF receptor JM region showed no cross-linking in the absence of ligand and 20–30% cross-linking efficiency upon EGF binding (31), consistent with a mechanism of ligand-induced dimerization and allosteric kinase activation (27, 28, 38–40).

**FIGURE 9.**
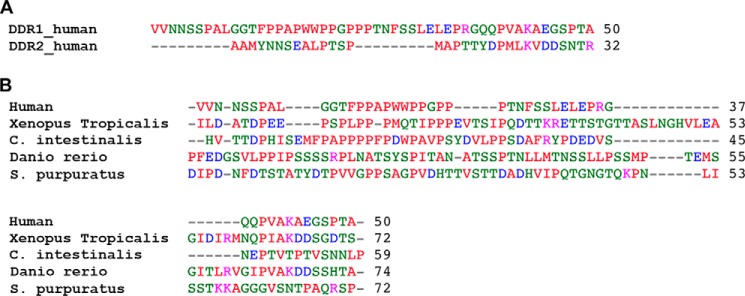
**Alignment of selected DDR1 and DDR2 JM sequences, performed with ClustalW 2. 1.** *A*, human DDR1 and DDR2. *B*, DDR1 from selected orthologues. Sequences are from *Homo sapiens* DDR1 Q08345; *Homo sapiens* DDR2 Q16832; *Xenopus tropicalis* (western clawed frog) XP_002933824; *Ciona intestinalis* (sea squirt) XP_002123027; *Danio rerio* (zebrafish) XP_001345829; *Strongylocentrotus purpuratus* (sea urchin) XP_001202828.

The insulin receptor is activated by ligand-induced conformational changes within a constitutive dimer (41). Could a similar mechanism apply to dimeric DDR1? There is currently no evidence that collagen binding to DDRs is associated with large conformational changes in the receptor (19, 23). Moreover, our new results demonstrate that the DDR1 JM region is very flexible, making it difficult to envisage how this region could transmit a conformational change. Disulfide formation requires the cysteine Cα atoms to be within ∼7 Å from each other, and the high dimerization efficiency of all our cysteine mutants in the JM region therefore indicates a highly flexible structure. The JM regions are not conserved between DDR1 and DDR2 or between DDR1 orthologues, but proline, glycine, or serine residues are abundant in all sequences (Fig. 9), suggesting that the JM region may function as a flexible linker in all DDRs. Proteolytic shedding of the DDR1 ectodomain has been described (21, 42, 43) and may require an unstructured JM region. A lack of rigidity within the JM region may also be required for efficient ligand binding or for interactions with other, as yet unidentified, proteins at the cell surface that might be involved in transmembrane signaling by DDRs.

Remarkably, covalent cross-links near the cell membrane and in the N-terminal part of the TM domain (residues 408–421) did not affect DDR1 function: the cysteine mutants did not have increased basal activity, nor did they have an attenuated response to collagen (Figs. 4 and 8). In sharp contrast, replacing JM residues with cysteine yielded constitutively active mutants for Erb-B2, the erythropoietin receptor, the p75 neurotrophin receptor and the natriuretic peptide receptor A (29, 30, 32–34) and common gain-of-function mutations in other RTKs result in unpaired cysteines that form intermolecular disulfide bonds (44–47). In the EGF receptor, extracellular insertion of a flexible sequence close to the cell membrane resulted in increased basal activation (28), whereas DDR1 activation was unaffected by deletions from the JM region and by insertions of flexible glycine-serine sequences (Figs. 7 and 8). The remarkable tolerance to drastic mutations within the proximal JM region distinguishes DDR1 from other RTKs and indicates an activation mechanism without conformational coupling across the plasma membrane. Clearly, ligand binding is somehow transmitted to the cytosolic region and leads to kinase activation, but our data suggest that the highly flexible JM region is unlikely to transmit a conformational change in DDR1.

In contrast to the proximal JM region, the distal JM region was more sensitive to enforced dimerization, with a disulfide cross-link at position 387 preventing activation by collagen. Covalent dimerization at two positions in the DS-like domain (V220C and L247C mutants) also strongly attenuated collagen-induced tyrosine phosphorylation. Thus, constraining the structure of the DDR1 dimer by disulfide cross-links farther away from the cell membrane interferes with the normal functioning of the receptor. Of note, we previously identified a patch on the DS domain that is required for signaling without being involved in collagen binding (19). Thus, it seems that the signaling state of DDR1 involves specific contacts between ectodomains farther away from the cell membrane that presumably cannot form in the P387C, V220C, and L248C dimers. To restrict the basal activity of DDR1, autoinhibitory mechanisms have to be in place which prevent these activating contacts in the absence of collagen. A recent study showed that mutation of the conserved glycosylation site at Asn-211 results in constitutive DDR1 phosphorylation (48). Asn-211 is located halfway between Val-220 and Leu-247 (Fig. 6*A*), suggesting that this region may indeed participate in forming the signaling state.

If neither ligand-induced dimerization nor conformational changes within a constitutive dimer can explain DDR1 activation, what other mechanism might be in operation? A plausible hypothesis is that collagen binding induces DDR1 clustering in the cell membrane. In this mechanism, constitutive DDR dimers could effectively function as the receptor units that are brought together in response to ligand binding. FRET experiments have indicated DDR1 clustering in response to collagen (16), but detailed studies using single-molecule techniques are currently lacking. Whereas our biochemical experiments show that DDR1 is dimeric in the absence of ligand, the data do not exclude higher order association states. Clustering is not without precedent in RTKs: Eph receptors initially bind ephrin ligands in a 2:2 complex (49), but higher order clustering in cell-cell contacts is required for receptor activation (50, 51). An attractive aspect is that a clustering mechanism might explain the slow activation kinetics of DDRs.

How could collagen binding trigger DDR clustering? Multivalency of (fibrillar) collagen is not required for DDR activation, given the observation that short triple-helical peptides elicit DDR phosphorylation with the same kinetics as native collagen (24, 25). Therefore, collagen binding must somehow change the structure of the DDR ectodomain in a way that favors clustering or that allows it to interact with another membrane protein that facilitates clustering. Whether this occurs by relieving an autoinhibited conformation or by bridging of DDR dimers by the ligand is currently unknown. The V220C, L247C, and P387C mutants might be inactive because the unnatural covalent dimers cannot bind collagen effectively or cannot interact laterally once collagen is bound. The inhibitory anti-DDR1 Abs described previously (19) may act similarly, by preventing the lateral association of collagen-bound DDR1 dimers within the plasma membrane. Finally, the constitutive activity of DDR1 lacking a glycan at position 211 (48) might be due to increased lateral association in the absence of collagen. Further studies are required to delineate the mechanism of the presumed clustering of DDRs.

## Footnotes

2 The abbreviations used are:

RTK: receptor tyrosine kinase
Ab: antibody
DDR: discoidin domain receptor
DS: discoidin homology
JM: juxtamembrane
NEM: *N*-ethylmaleimide
TM: transmembrane.
